# Selenium Nanoparticles Can Influence the Immune Response Due to Interactions with Antibodies and Modulation of the Physiological State of Granulocytes

**DOI:** 10.3390/pharmaceutics14122772

**Published:** 2022-12-11

**Authors:** Venera V. Khabatova, Dmitriy A. Serov, Irina V. Tikhonova, Maxim E. Astashev, Egor I. Nagaev, Ruslan M. Sarimov, Tatiana A. Matveyeva, Alexander V. Simakin, Sergey V. Gudkov

**Affiliations:** 1 Prokhorov General Physics Institute of the Russian Academy of Sciences, 38 Vavilove St., 119991 Moscow, Russia; 2 Federal Research Center “Pushchino Scientific Center for Biological Research of the Russian Academy of Sciences”, Institute of Cell Biophysics of the Russian Academy of Sciences, 3 Institutskaya St., 142290 Pushchino, Russia

**Keywords:** selenium nanoparticles, NPs fragmentation, SeNPs, granulocytes, cytotoxicity, antibody binding to NPs

## Abstract

Currently, selenium nanoparticles (SeNPs) are considered potential immunomodulatory agents and as targets for activity modulation are granulocytes, which have the most abundant population of immune blood cells. The present study aims to evaluate the cytotoxic effect and its effect on the functional responses of granulocytes. In addition to the intrinsic activity of SeNPs, we studied the activity of the combination of SeNPs and IgG antibodies. Using laser ablation and fragmentation, we obtained nanoparticles with an average size of 100 nm and a rather narrow size evolution. The resulting nanoparticles do not show acute toxicity to primary cultures of fibroblasts and hepatocytes, epithelial-like cell line L-929 and granulocyte-like culture of HL-60 at a concentration of 10^9^ NPs/mL. SeNPs at a concentration of 10^10^ NPs/mL reduced the viability of HL-60 cells by no more than 10% and did not affect the viability of the primary culture of mouse granulocytes, and did not have a genotoxic effect on progenitor cells. The addition of SeNPs can affect the production of reactive oxygen species (ROS) by mouse bone marrow granulocytes, modulate the proportion of granulocytes with calcium spikes and enhance fMLF-induced granulocytes degranulation. SeNPs can modulate the effect of IgG on the physiological responses of granulocytes. We studied the expression level of genes associated with inflammation and cell stress. SeNPs increase the expression of catalase, NF-κB, Xrcc5 and some others; antibodies enhance the effect of SeNPs, but IgG without SeNPs decreases the expression level of these genes. This fact can be explained by the interaction between SeNPs and IgG. It has been established that antibodies interact with SeNPs. We showed that antibodies bind to the surface of selenium nanoparticles and are present in aqueous solutions in a bound form from DLS methods, ultraviolet–visible spectroscopy, vibrational–rotational spectrometry, fluorescence spectrometry, and refractometry. At the same time, in a significant part of the antibodies, a partial change in the tertiary and secondary structure is observed. The data obtained will allow a better understanding of the principles of the interaction of immune cells with antibodies and SeNPs and, in the future, may serve to create a new generation of immunomodulators.

## 1. Introduction

Selenium is known to be an essential chemical element for living organisms. The human body is believed to contain 10–14 mg of selenium. The daily human need for selenium is 70–100 µg [1]. Selenium is involved in the regulation of the metabolism and the maintenance of redox homeostasis. The amino acid selenocysteine is a part of the active centers of many enzymes in the form of [2]. Selenium is used for the effective absorption of iodine [3], as an antioxidant micro-additive [4], radioprotector [5] and radiomitigator [6]. Selenium deficiency is known to affect the functioning of the immune system [7]. It has been shown that a normalized intake of selenium is important for the functioning of most cells of innate immunity [8,9]. The immunomodulatory properties of selenium are likely partly associated with this chemical element’s antioxidant properties. In many scenarios, selenium can prevent the development of oxidative stress and its detrimental consequences, including damage to nucleic acids, proteins, and other biomolecules [10,11]. In addition, selenium is part of 25 selenoproteins that play an important role in the regulation of the Red/Ox potential and the functioning of immune cells [12].

Currently, the researchers’ interest is focused on the nanosized forms of selenium (selenium nanoparticles (SeNPs) and nanofibers). Selenium nanoparticles are widely used for biomedical applications [13] and agriculture [14]. Typically, SeNPs consist of nonoxidized selenium Se^0^. Se^0^ is insoluble in water, and is slowly oxidized and transformed into selenium oxides. Oxides of selenium are already highly soluble in water and can quickly enter into biological cycles, but selenium oxide concentrations are not toxic [15]. That is why selenium nanoparticles have low toxicity and can have a powerful, prolonged effect on living systems [16]. It has been shown that SeNPs are much more toxic to cancer cells compared to normal ones [17]. In addition, SeNPs can potentially adsorb drug molecules on their surface and are chemically stable. Therefore SeNPs are good candidates for manufacturing drug delivery vehicles or biomacromolecules for chemotherapy [18]. The SeNPs with 50–100 nm size is more preferred for use because they are not absorbed by the reticuloendothelial system and are not filtered out in the kidneys [16].

SeNPs can modulate the functioning of the immune system, for example, and the production of cytokines by macrophages [19]. SeNPs may inhibit tumor growth and development by activating the specific T cells and tumor-associated macrophages [20]. SeNPs are also being considered as an agent for preventing immunosuppression in chemotherapy. The administration to the mice SeNPs capped with β-glucan stimulated the production of immune factors by immune cells in the murine model of cytoxan-induced immunosuppression [21]. Currently, immunosuppression during chemotherapy seriously increases the risk of infectious diseases in cancer patients. Moreover, the infections can be caused by commensal potentially pathogenic microorganisms. In general, there are sufficient numbers of studies in which SeNPs are considered a universal new therapeutic agent for neutralizing immune system dysfunction in bacterial infections, cancer, and other diseases associated with immunosuppression [22,23,24].

Granulocytes are a subgroup of white blood cells with a large segmented nucleus and specific granules in the cytoplasm. Neutrophilic, eosinophilic, and basophilic granulocytes are defined depending on the staining of specific granules [25]. Usually, specific granules are large lysosomes, peroxisomes or modifications of these organelles [26]. Granulocytes are key effector cells of the innate immune system. They provide primary protection against pathogens and eliminate virus-infected or transformed (mutant and cancer) host cells. In addition, the granulocytes are involved in the regulation of inflammation [27]. Granulocytes use direct and indirect mechanisms for pathogen elimination. Direct mechanisms include respiratory burst (generation of reactive oxygen species (ROS)), release of neutrophil extracellular traps (NETs), and phagocytosis [28]. Indirect mechanisms are mainly associated with producing a large number of signal molecules, including cytokines [29]. Granulocytes can migrate to the area of damage and inflammation [30]. In some autoimmune and oncological diseases, the activity of granulocytes can pose serious harm to the patient’s body due to excessive cytotoxic activity, generation of ROS, malfunctioning of the NET system and the development of chronic inflammation [31,32]. The activity of the granulocytes is regulated by both small signal molecules and proteins, including antibodies [33,34,35]. On the surface of granulocytes, six FcγR subtypes are expressed, through which antibodies can trigger PKC-, PLCγ-, PI3K- and ERK-dependent intracellular signaling pathways that regulate such granulocyte functions as cytokine secretion, ROS production, cytoskeletal rearrangement and NETosis [36,37,38]. The literature describes the ability of selenium to influence signal transduction along intracellular signaling pathways, including those involving PKC and PI3K [39,40,41]. However, the effects of the combined action of selenium and antibodies on the functioning of innate immunity cells remain poorly understood.

This study is mainly focused on the modulation of the physiological state of granulocytes by SeNPs, as well as the ability of SeNPs to influence the immune status through interaction with antibodies.

## 2. Materials and Methods

### 2.1. SeNPs Fabrication and Characterisation

SeNPs were fabricated from a solid Se^0^ target (Sigma Aldrich, Burlington, Massachusetts, USA) by laser ablation with subsequent fragmentation in water with 1060–1070 nm Ytterbium-doped fiber laser (YLPM-1). Laser pulses had an average power of 20 mV, duration of 80 ns and frequency of 20 kHz. The characteristics of acoustic oscillations (breakdown shockwave amplitude) and generated plasma (average distance between optical breakdowns in a track) were registered in the experimental cell during laser fragmentation to examine NPs size evolution. After fragmentation, the hydrodynamic diameter and ζ-potential of obtained SeNPs were measured with Zetasizer Ultra Red Label (Malvern Panalytical, Malvern, UK). The diameter of dried SeNPs was evaluated with a Libra 200 FE HR transmission electron microscope (Carl Zeiss, Oberkochen, Germany). More detailed descriptions can be found in previous works [42,43].

### 2.2. Animal Study

The study was carried out on male mice of the BALB/c line weighing 21–24 g (8–10 weeks). The animals were purchased from the “Stolbovaya” Branch of the Scientific Biomedical Technology Center of the Federal Medico-Biological Agency (Moscow region, Russia). All procedures with animals were performed following the European Communities Council Directive (24 November 1986; 86/609/EEC) and the Declaration on Humane Treatment of Animals. All experiments were carried out following the regulatory legal act of the Ministry of Health of the Russian Federation No. 199-n “On approval of the rules of good laboratory practice, international legal norms specified in the European Convention ETS No. 123 “On the protection of vertebrate animals used for experiments or in other scientific purposes”. The animal study protocol was approved by the Institutional Ethics Committee of the Institute of Cell Biophysics of the Russian Academy of Sciences (12306, 2006). Animals received drink and food *accesso libero*.

### 2.3. Isolation of Granulocytes

The isolation of granulocytes from mouse bone marrow was performed by centrifugation on a Percoll density gradient according to the standard method [44]. Mice were immobilized by cervical dislocation. The femur, tibia, and ulna were removed, the epiphyses were cut off, and washed with RPMI-1640 medium (Gibco, Waltham, MA, USA). The cell suspension was layered on a Percoll gradient in PBS (78%, 62.5%, 55%, *v/v*) and centrifuged at 1500× *g*, 35 min, 4 °C. Cells were harvested from the 62.5% and 78% boundary, washed in RPMI-1640, then in PBS, and diluted in HBSS without calcium to a concentration of 10^7^ cells/mL. Cells were kept for 1 h on ice for resting. The purity and viability of isolated cells were evaluated by fluorescence microscopy, described below. The viability of the isolated cells was assessed with trypan blue staining. Mature granulocytes have a polymorphic nucleus and high level of Gr-1 receptor expression. The polymorphic form of the nucleus was confirmed by staining with Hoechst 33258. The Gr-1 expression level was assessed by staining with PE-conjugated monoclonal anti-Ly-6G/Ly-6C (Gr-1) antibodies clone (Thermo Fisher, Waltham, MA, USA). The evaluation was performed using a DM 6000 fluorescent microscope (Leica, Wetzlar, Germany). Only samples of isolated cells with a survival rate of at least 98% and a proportion of polymorphonuclear Gr-1^+^ cells of at least 90% were taken in an experiment.

### 2.4. Evaluation of ROS Production

ROS production was assessed with the chemiluminescent method using a Chemilyum-12 chemiluminometer (ICB RAS, Pushchino, Russia). Cells (10^6^ cells/mL) were incubated in disposable measuring cells (Institute for Biological Instrumentation of the Russian Academy of Sciences, Pushchino, Russia) for 20 min at 37 ℃ in the presence of 0.35 mM luminol, 0.1 mM NaN_3_, 1 unit/mL horseradish peroxidase type IV, pH 7.38. After the incubation, the samples were placed in the measuring chamber of the chemiluminometer. The baseline luminescence level was recorded for 4 min, then the corresponding stimulus 10^10^ SeNPs and/or 500 μg/mL polyclonal IgG (Minigene, Russia) was added. Next, the luminescence was recorded for 20 min, after which the respiratory burst inducers were added: 1 μM of bacterial protein mimetic synthetic peptide WKYMVM (Wp) or the PKC activator 1 µM PMA [45,46]. The intensity of cell chemiluminescence was recorded for 250 s. Each sample was measured in duplicate. A more detailed description of the technique is given in [47].

### 2.5. Assessment of Calcium Responses of Cells

The concentration of calcium ions in the cytosol ([Ca^2+^]_i_) of mouse granulocytes was assessed using fluorescence microscopy. A cell suspension (10^6^ cells/mL) in the form of a drop of 100 µL was applied to a round coverslip (d 25 mm), placed in a humid chamber, and incubated for 15 min at 37 °C for its attachment. Next, the cells were stained with a Fura2-AM fluorescent probe (Thermo Fisher, USA) for 45 min at 37 °C in a humid chamber. The slides with cells were mounted in a cover slip holder (RC-40LP, Warner Instruments, Hamden, CT, USA). Cells were immediately washed twice with 1 mL of complete HBSS preheated to 37 °C, 1 mL of heated HBSS was added to the washed cells and placed on a thermostatically controlled (37 °C) stage. Fluorescent signals were recorded using an imaging setup based on the AE31E interposed microscope (Motic, Barcelona, Spain), the SDU-285 digital camera (SpecTeleTechnika, Moscow, Russia), and the LED illumination system. Fura2-AM fluorescence was recorded in the ratiometric mode at excitation wavelengths of 340 nm and 380 nm. The calcium concentration in the cytoplasm of granulocytes ([Ca^2+^]_i_) was estimated from the F340/F380 fluorescence ratio with preliminary subtraction of background values [48]. The settings for the LED illuminator current, digital camera exposure time, and gain were constant for all experiment variants.

Cell fluorescence was recorded under basic conditions for 4 min, then 0.9 × 10^10^/mL SeNPs, 500 μM IgG, or their combination was added, and cell fluorescence was recorded for 15 min. Then, the pro-inflammatory agent W-peptide (1 μM) was added, and calcium responses were recorded within 5 min. Sterile PBS was used as the first additive in the control samples. WinFluorXE acquisition software (J. Dempster, Strathclyde Electrophysiology Software, University of Strathclyde, UK) was used for data collection. A more detailed description of the measurement setup and data acquisition mode can be found in the previous study [49].

The calcium activity of cells was assessed by the proportion of cells with calcium responses in the total population after each addition. This approach is based on the calculation of the third-order statistical moment (asymmetry, As) of the F340/F380 values for each cell under each of their experimental conditions. The research team developed this method earlier to assess the calcium responses of electrically nonexcitable cells. A detailed description of the principle of the method can be found in [50].

### 2.6. Micronucleus Test

Cytogenetic cell damage was assessed by the appearance of polychromatophilic erythrocytes (PCEs) containing micronuclei (MN). The maximum yield of PCE with MN is observed approximately one day after exposure; therefore, the samples were prepared 24 h after the addition of nanoparticles. The samples for the microscope were prepared and stained according to the method [51]. PCE-containing MN were counted using a light microscope with an immersion lens at a magnification of ×1000. Details were described in the published research [52].

### 2.7. Cytotoxicity Assay

Acute cytotoxicity studies were performed using four types of cell cultures as test systems in vitro primary cultures of mouse hepatocytes, bone marrow granulocytes, fibroblasts, as well as permanent cell lines L-929 (mouse connective tissue, ATCC NoCCL-1) and HL-60 (human leukemia cells, ATCC NoCCL-240). For routine procedures with cells, we used DMEM/F12 medium (1:1), fetal bovine serum (FBS), L-glutamine, penicillin-streptomycin, 0.05% trypsin-EDTA solution, purchased from the PanEco Company (PanEco, Moscow, Russia). All reagents used in the experiments were of analytical purity. Cells were cultured in T-25 flasks (TPP, Trasadingen, Switzerland) at 37 °C and 5% in an S-Bt Smart Biotherm CO_2_ incubator (Biosan Riga, Latvia). For the cultivation of primary cell cultures and line L-929, we used DMEM/F12 medium supplemented with 10% FBS, 2 mM L-glutamine, 100 U/mL penicillin, and 100 μg/mL streptomycin. The HL-60 cell line was cultivated in DMEM/F12 medium containing 20% FBS. The medium was replaced with a freshly prepared one every 2 days. Before the experiment, the cells were detached from the surface of the culture flask using a 0.05% trypsin-EDTA solution. Trypsin was inactivated with 10% FBS solution, and the cells were centrifuged at 350 g for 5 min. Cells were seeded into wells of 12-well plates (TPP, Switzerland) before experiments. Murine granulocytes were cultured in RPMI1640 medium supplemented with 10% FBS, 2 mM L-glutamine, 100 U/mL penicillin, and 100 μg/mL streptomycin (PanEco, Moscow, Russia). Cells were incubated with different concentrations of SePNs (10^7^–10^11^ NPs/mL) for 24 h for the granulocytes and 72 h for the other cells in the CO_2_ incubator. After incubations, the cells were stained with 2 μg/mL Hoechst 33258 (Thermo Fisher, USA) to visualize nuclei of all cells (alive + dead) for 30 min, washed with BPS and stained with 2 μM propidium iodide (dead only) during 1 min (Thermo Fisher, Massachusetts, USA). The fluorescence intensities of cells were evaluated with a DMI6000 microscope (Leica, Munchen, Germany). The data were analyzed with ImageJ2 (Fiji) software (NIH, Bethesda, USA). Additionally, the areas of cells nuclei were calculated with automatic procedures “threshold”, “analyze particles” and “measure area”. At least 200 cells were analyzed in each sample. For each variant of the experiment, at least three samples were analyzed.

### 2.8. Gene Expression

Real-time RT PCR was applied to analyze gene expression in surviving mouse granulocyte cultures. Total RNA from cells was extracted using ExtractRNA reagent. The quality of RNA was assessed by electrophoresis in 2% agarose gel in TAE buffer in the presence of ethidium bromide (1 µg/mL). The RNA concentration was measured on a NanoDrop 1200c spectrophotometer (Metler Nolledo, Greifensee, Switzerland). To avoid possible contamination with genomic DNA, the isolated RNA was treated with RQ1 DNAse. For reverse transcription (RT), 2 µg of total RNA was used with an MMLV RT kit. The obtained cDNA was subsequently used in PCR with gene-specific primers (Table 1) synthesized by Evrogen (Moscow, Russia). Real-time PCR was run in thermocycler QuantStudio 5 (Thermo Fisher Scientific, USA) using a qPCRmix-HS kit, which contains fluorescent intercalating dye SYBR Green II. Results were calculated according to a standard method. All details have been previously described [53].

### 2.9. Degranulation Assay

Mouse bone marrow granulocyte degranulation was assessed by staining with a LysoTracker fluorescent probe [54]. One pre-sterilized round coverslip, 25 mm in diameter, was placed in each well of a six-well plate. The slides were coated with a suspension of isolated granulocytes (100 μL of complete Hank’s solution with 10^7^ cells/mL and incubated for 15 min at 37 °C in a humid chamber for cell attachment. Then, 1 mL of complete Hank’s solution was added to each cell with glass. SeNPs and/or IgG were added to the corresponding wells at final concentrations of 10^10^ NPs/mL and 0.5 mg/mL, respectively. Next, the cells were incubated for 20 min at 37 °C in a humid chamber, and 1 μM fMLF was added to the corresponding cells and incubated for 15 min under the same conditions. After incubation, the cells were washed twice with Hank’s solution and placed on ice. Immediately prior to analysis, cells were stained with 50 nM LysoTracker Green (Thermo Fisher, USA) for granule visualization and NucRed (Thermo Fisher, USA) for nuclear visualization. Confocal images were obtained using a DMI6000 microscope (Leica, Germany). The degree of degranulation was assessed by the depletion of the cytoplasmic pool of granulocytes. Analysis was performed using Image J2 (Fiji) software (NIH, USA). Only segmented cells were analyzed. For each variant of the experiment, at least six samples were analyzed; in each of those, at least 20 cells were analyzed.

### 2.10. Optical Research Methods

Absorption spectra were measured on a Cintra 4040 (GBC Cintra 4040, Australia) in quartz cuvettes with an optical path length of 10 mm at room temperature (~22 °C). The BSA concentration was 0.5 g/L. The absorption spectra were measured with six to eight samples for each group. Zetasizer ULTRA Red Label (Malvern Panalytical Ltd., Malvern, UK) was used to obtain information on hydrodynamic particle diameters. A 1 mL solution of lysozyme with a concentration of 0.4 mg/mL was measured in a plastic cuvette at 25 °C. Five independent experiments were carried out for the control and each point of influence. The intensity distributions of the hydrodynamic diameters were calculated using the ZS Xplorer program and algorithm [55]. The fluorescence of samples in water was studied on a Jasco FP-8300 spectrometer (JASCO Applied Sciences, Nova Scotia, Canada). Measurements of a 2 mL solution of IgG with a protein concentration of 5 g/L were carried out in quartz cuvettes with an optical path length of 10 mm at room temperature (~25 °C). Each sample was measured three times. The figures show typical spectra; with repeated measurements, the intensity maxima change by several percent [56]. Refractive index measurements were carried out on a Multiwavelengths Refractometer: Abbemat MW (Anton Paar, Graz, Austria). In the experiments, 1 mL of the solution was poured into the cell of the device and measurements were made at a wavelength of 435.8, 589.3 and 632.8 nm at a temperature of 25 °C [57].

### 2.11. Statistics

Data processing was performed with Origin (OriginLab Corporation, Massachusetts, USA) and SigmaPlot (Systat Software, Palo Alto, USA) software. All data are represented as means ± standard error of the mean. The significance of differences between the samples was assessed by the Mann–Whitney test or Kruskal–Wallis one-way analysis of variance on ranks with multiple pairwise comparisons by Tukey’s Test in the case of independent samples. One sample signed-rank test Z-statistic (based on positive ranks) was used to compare normalized data with the control.

## 3. Results

**Figure 1:**
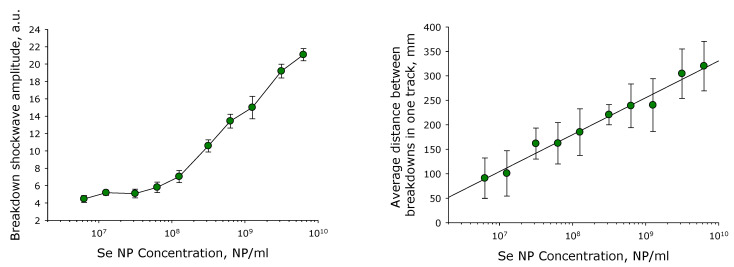
The effect of Selenium Nanoparticles Concentration on the Amplitude of Acoustic Oscillations and the Average Distance between Optical Breakdowns during Laser Fragmentation. Data are presented as means ± standard error of the mean. The three independent experiments were carried out for each variant (*n* = 3).

It is shown that the change in the amplitude of acoustic oscillations can be described by two linear functions in the coordinates presented (Figure 1). The first is in the concentration range from 5 × 10^6^ to 10^8^ NPs/mL. The second is in the concentration range from 10^8^ to 10^10^ NPs/mL. In this case, the interpolation accuracy will be quite high due to the small measurement variability. It has been established that the optical changes in the experimental cell during laser fragmentation can be described by one linear function in the coordinates presented and not by two functions, as is usually the case. Therefore, the interpolation accuracy will be somewhat lower.

**Figure 2:**
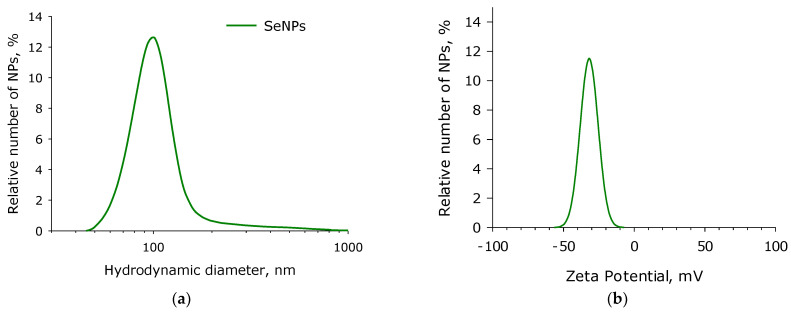

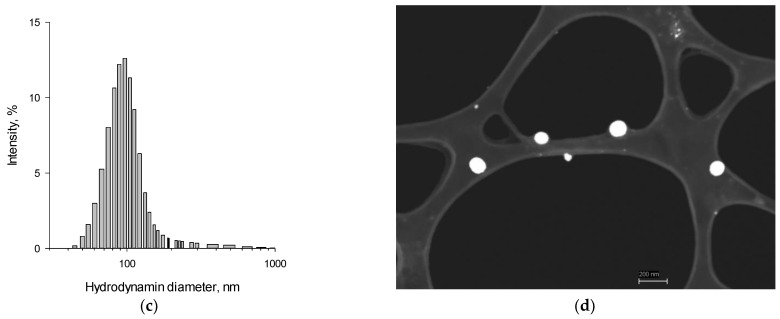
SeNPs characteristics: (**a**) size distribution with the use of dynamic light scattering; (**b**) ζ-potential distribution; (**c**) size distribution with the use of transmission electron microscopy; (**d**) example of TEM pictures. The scale bar is 200 nm.

The resulting nanoparticles were studied using the dynamic light scattering method. Thus, by controlling the physicochemical processes in the experimental cell, we managed to obtain nanoparticles with a rather narrow size evolution (Figure 2a). It is shown that the average hydrodynamic diameter of nanoparticles is close to 100 nm. The distribution half-width is about 35 nm (from 70 to 105 nm). TEM data confirm these results (Figure 2c,d). The average diameters of dried SeNPs were 94–96 nm. The ζ-potential of SeNPs were about ~30 mV (Figure 2b).

**Figure 3:**
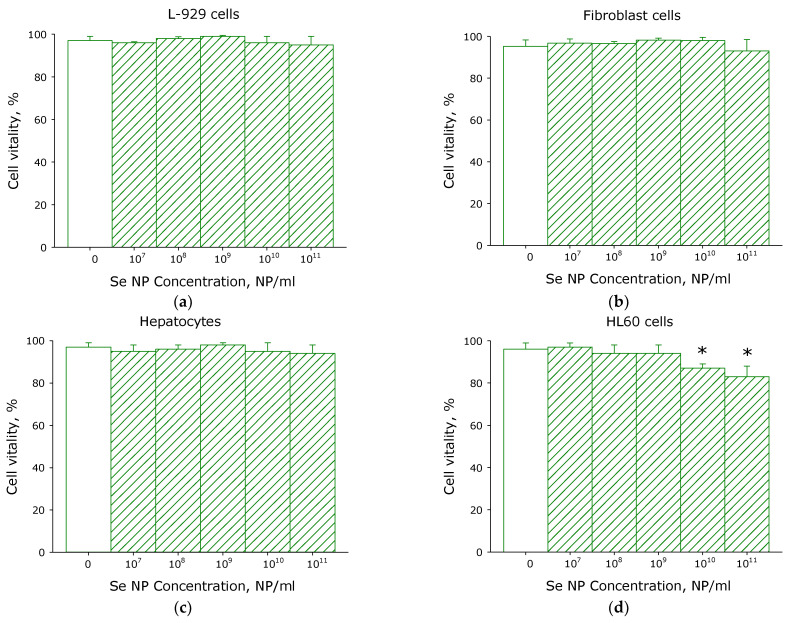
The effect of selenium nanoparticles in different concentrations on the survival of cells of various origins: L-929 cells (**a**), fibroblasts (**b**), hepatocytes (**c**), HL60 cells (**d**). Data are presented as means ± standard error of the mean. The three independent experiments were carried out for each variant (*n* = 3). *—difference from the control values (*p* < 0.05). The significance of differences was assessed by the Mann–Whitney test.

The effect of selenium nanoparticles on the viability of normal and immortalized cells was studied (Figure 3). It has been shown that selenium nanoparticles do not affect the viability of fibroblasts and hepatocytes, as well as the epithelial-like cell line L-929. When studying the HL-60 leukemia cell line (granulocyte-like culture), it was found that at a concentration of selenium nanoparticles of 10^10^ and 10^11^ PNs/mL, cell survival decreases by 9% and 13%, respectively. When assessing survival, various indicators were calculated programmatically, including the area of the nucleus. It was found that with an increase in the concentration of selenium nanoparticles to 10^11^ PNs/mL in the L-929 cell culture, the area of the nucleus decreases by slightly more than 15%. In the HL60 culture, a statistically distinct compaction of the nucleus is already observed at a concentration of about 10^9^ PNs/mL of selenium nanoparticles. At high concentrations (10^10^ and 10^11^ PNs/mL) of nanoparticles, the area decreases by 20–30%.

**Figure 4:**
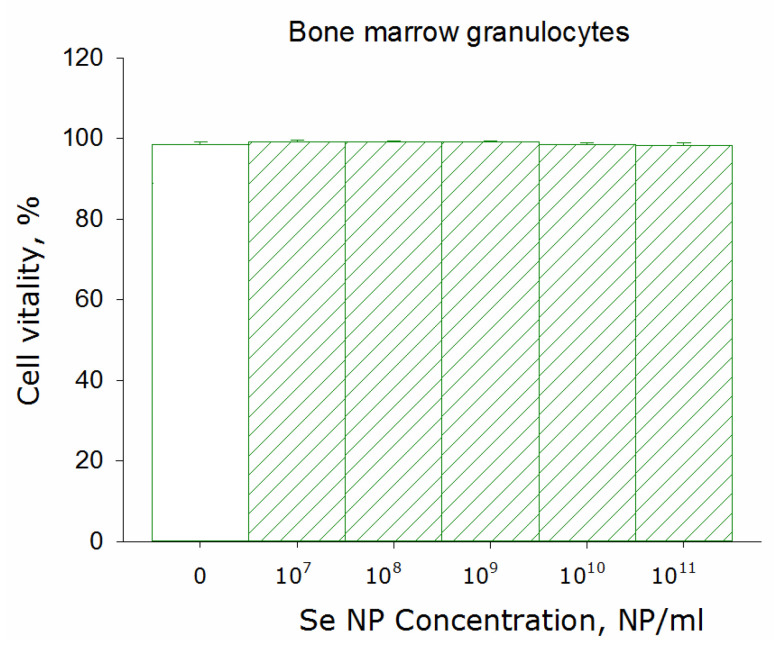
The effect of selenium nanoparticles in different concentrations on the survival of murine bone marrow granulocytes after 24 h incubation. Data are presented as means ± standard error of the mean. The three independent experiments were carried out for each variant (*n* = 3).

Thus, SeNPs have been shown to affect granulocyte-like culture HL-60 viability. When conducting a similar study on a surviving granulocyte culture, it was shown that the addition of selenium nanoparticles has a more significant effect. During incubation of cells with SeNPs at a concentration of 10^8^ PNs/mL, a tendency to viability decreasing was observed. The HL-60 viability decreases by 10–20% at SeNPs concentrations of 10^10^ and 10^11^ NPs/mL. The experiments were carried out on a surviving culture of mouse granulocytes. It was shown that no loss of viability was observed even at a concentration of 10^11^ nanoparticles per mL (Figure 4). Therefore, SeNPs do not have cytotoxic activity against normal primary granulocytes but decreased viability of the myeloid leukemia HL-60 cell line. The granulocytes should be fairly resistant to environmental changes. 

The question arises whether nanoparticles can affect progenitor cells (red bone marrow cells) (Table 2). Using the micronucleus test, it was shown that selenium nanoparticles up to a concentration of 10^10^ PNs/mL do not affect the formation of micronuclei, although there is a tendency to increase the number of cells containing micronuclei. For example, the percentage of cells containing micronuclei increases by 12% compared to the control at a concentration of selenium nanoparticles of 10^10^ PNs/mL. At a concentration of 10^11^ selenium nanoparticles, the number of cells containing micronuclei significantly increases by almost 30%. Thus, selenium nanoparticles can affect normal granulocytes, while granulocytes and myeloid progenitor cells of granulocytes are quite resistant to the action of selenium nanoparticles.

**Figure 5:**
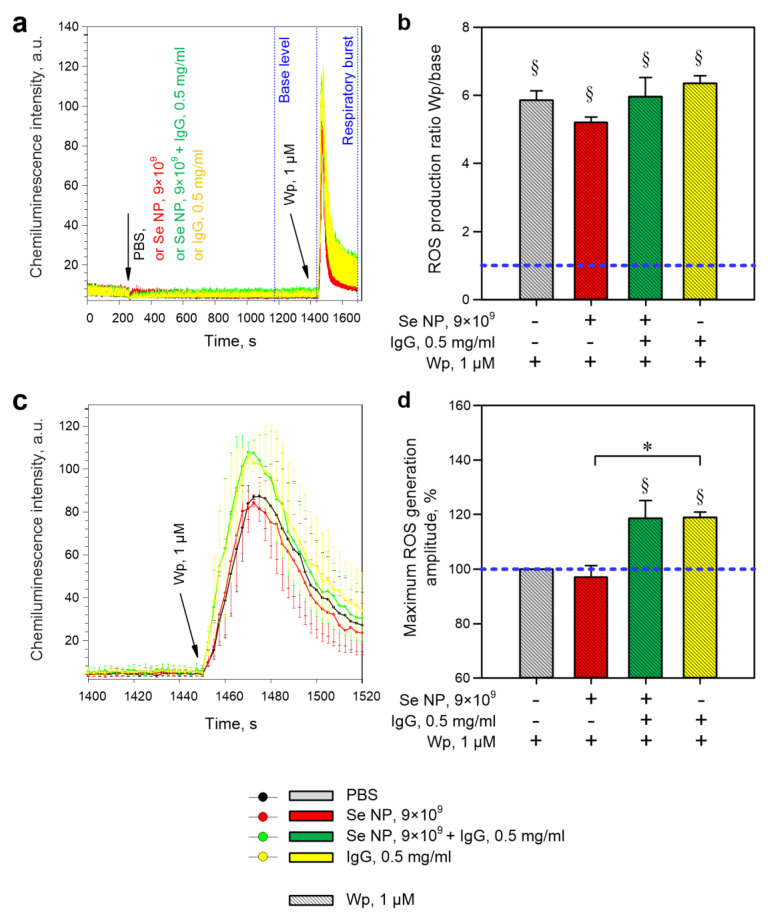
The evaluation of ROS production by mouse bone marrow granulocytes. (**a**) Full average records of the dynamics of chemiluminescence intensity in different variants of the experiment. The times of addition of the respective substances are indicated with arrows. The time intervals for which the “base level” and Wp-induced “respiratory burst” ROS production is calculated are shown in the figure with dotted blue lines with the corresponding labels. (**b**) Average ROS production calculated as the ratio of the integral of the chemiluminescence intensity 250 s after the addition of Wp to the integral of the chemiluminescence intensity 250 s before the addition of the stimulus (taken as one blue horizontal line). (**c**) Fragments of the averaged records of the dynamics of chemiluminescence intensity in different variants of the experiment (only the beginning of the response to 1 μM Wp). (**d**) Maximum amplitudes of ROS generation by mouse granulocytes calculated as the maximum intensity of chemiluminescence and expressed as a percentage where the maximum intensity of chemiluminescence in control is taken as 100% (blue horizontal lineAll data are presented as a mean ± SE. The four independent experiments were carried out for each variant (*n* = 4). §—*p* < 0.05 vs. PBS and 1 μM Wp, a one-sample signed-rank test Z-statistic (based on positive ranks). *—*p* < 0.05, Kruskal–Wallis one-way analysis of variance on ranks with a multiple pairwise comparison by Tukey’s Test.

**Figure 6:**
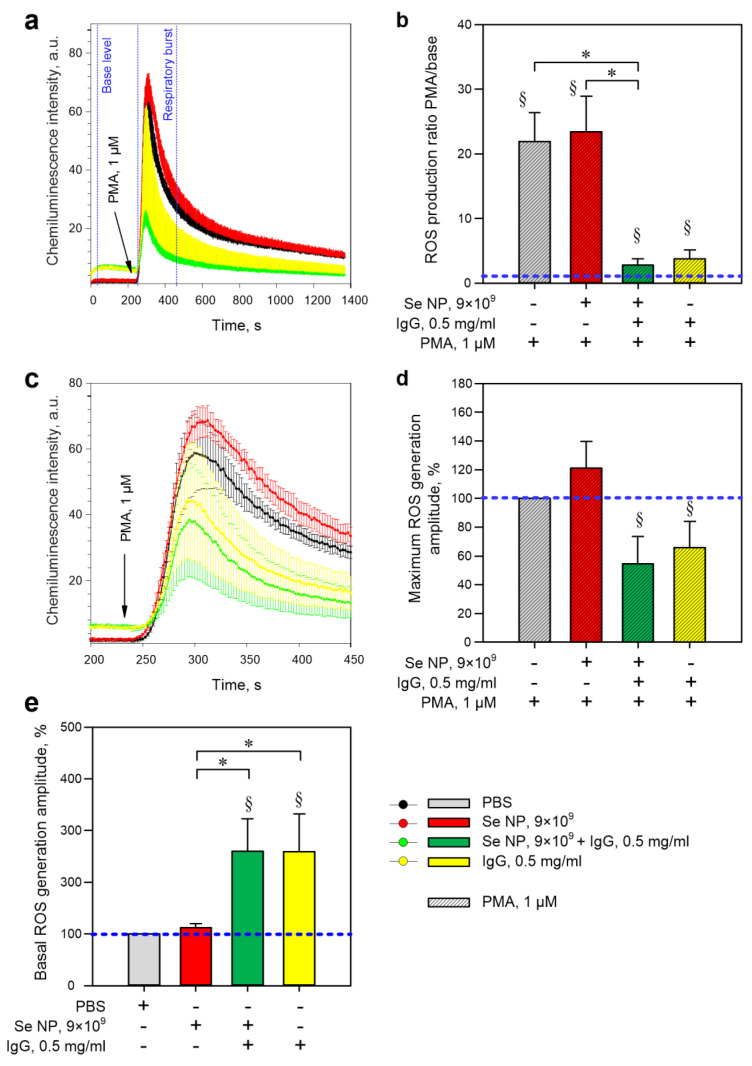
The evaluation of ROS production by mouse bone marrow granulocytes. (**a**) Full average records of the dynamics of chemiluminescence intensity in different variants of the experiment. The times of addition of the respective substances are indicated with arrows. The time intervals for the “base level” and PMA−induced “respiratory burst” ROS production were calculated and shown in the figure with dotted blue lines with the corresponding labels. (**b**) Average ROS production is calculated as the ratio of the integral of chemiluminescence intensity 250 s after PMA addition to the integral of chemiluminescence intensity 250 s before stimulus addition (taken as one, blue horizontal line). (**c**) Fragments of the averaged records of the dynamics of chemiluminescence intensity in different variants of the experiment (only the beginning of the response to 1 μM Wp). (**d**) Maximum amplitudes of ROS generation by mouse granulocytes, calculated as the maximum intensity of chemiluminescence, expressed as a percentage, where the maximum intensity of chemiluminescence in control is taken as 100% (blue horizontal line). (**e**) Total ROS production 250 s prior to the addition of 1 μM Wp or 1 μM PMA, where the total production in control (PBS) is taken as 100%. All data are presented as a mean ± SE. The four independent experiments were carried out for each variant (*n* = 4). §—*p* < 0.05 vs. PBS and 1 μM PMA, a one-sample signed-rank test Z-statistic (based on positive ranks). *—*p* < 0.05, Kruskal–Wallis one-way analysis of variance on ranks with a pairwise multiple comparison by Tukey’s Test.

It is known that granulocytes are cells of nonspecific immunity as a kind of first line of defense of the body. The main functions of granulocytes are the neutralization of microorganisms by generating ROS in the focus of infection. In this regard, the effect of selenium nanoparticles on the rate of ROS production by mouse bone marrow granulocytes was studied. Recently, a lot of information has appeared in the literature regarding the interaction of granulocytes with antibodies; therefore, the total fraction of the IgG antibodies was used together with selenium nanoparticles. It has been shown that adding selenium nanoparticles or antibodies (IgG) to granulocytes does not increase the production of ROS (Figure 5 and Figure 6). SeNPs do not modify the respiratory burst of granulocytes induced by Wp (Figure 5b,d) or PMA (Figure 6b,d). 

Furthermore, the addition of IgG also does not affect the total production of ROS (Figure 5b) but increases the maximum amplitude of the response (Figure 5d). The combination of IgG and SeNPs also increased the maximum amplitude of ROS generation in response to Wp, though it did not change the total ROS production in 250 s (Figure 5b,d). The presence of selenium nanoparticles did not modify the effects of IgG. Selenium nanoparticles did not affect the maximum amplitude and total production of ROS by granulocytes in response to PMA (Figure 6b,d). The addition of SeNPs + IgG or IgG alone caused a decrease in total ROS production in response to PMA (Figure 6c,d), though it did not affect the maximum amplitude of the response (Figure 6a,b). The presence of SeNPs did not modify the PMA-induced “respiratory burst” in the presence of IgG. In addition, IgG or SeNPs + IgG supplementation independently increased general ROS production (Figure 6c,e). However, as with PMA, no synergistic effects were observed between SeNPs and IgG, suggesting that the effect is due to IgG in both cases.

**Figure 7:**
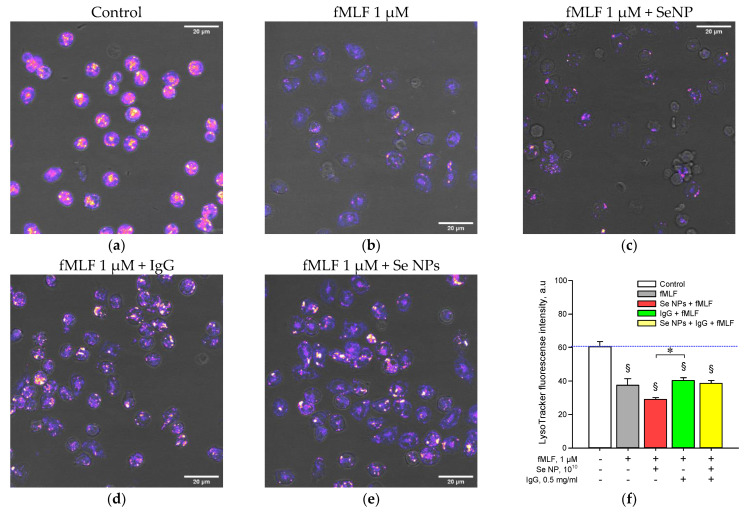
Effect of SeNPs and antibodies on the degree of fMLF-induced degranulation of mouse granulocytes. Micrograph examples: (**a**) control; (**b**) cells stimulated for 15 min with 1 μM fMLF; (**c**) Cells stimulated with 15 min 1 μM fMLF in the background of 20 min preincubation with 10^10^ SeNPs/mL; (**d**) cells stimulated with 1 μM fMLF for 15 min followed by 20 min of preincubation with 0.5 mg/mL IgG; (**e**) Cells stimulated with 15 min of 1 μM fMLF followed by 20 min of preincubation with a combination of 0.5 mg/mL IgG and 10^10^ SeNPs/mL. (**f**) Fluorescence intensity of LysoTracker in the cytoplasm of cells in different variants of the experiment. All data are presented as a mean ± SE. The four independent experiments were carried out for each variant (*n* = 4). §—*p* < 0.05 vs. control without stimulus, one-sample signed-rank test Z-statistic (based on positive ranks). *—*p* < 0.05, Kruskal–Wallis one-way analysis of variance on ranks with pairwise multiple comparison by Tukey Test. All images were obtained at similar settings of laser power, gain and pine hole. The equal dynamic range for the lookup table for LysoTracker was applied in all images. The equal dynamic range for the lookup tables was applied in all images. The photomicrographs are presented as an overlay of images in the visible region (gray) and pronounced LysoTracker fluorescence (table selection “fire”).

We investigated the effect of SeNPs on mouse granulocyte degranulation induced by 1 μM fMLF (Figure 7). The degree of degranulation was assessed by the intensity of LysoTracker fluorescence. We found that the addition of 1 μM fMLF caused a decrease in LysoTracker fluorescence by 35% compared to the control (Figure 7a,b). The pre-supplementation of 10^10^ NPs/mL SeNPs enhanced the fMLF-induced decrease in fluorescence intensity (up to 50% compared to the control). The introduction of IgG did not modify the effect of fMLF, but blocked the effect of SeNPs (Figure 7d,e). The data obtained may indirectly indicate the ability of IgG to block the effects of SeNPs.

**Figure 8:**
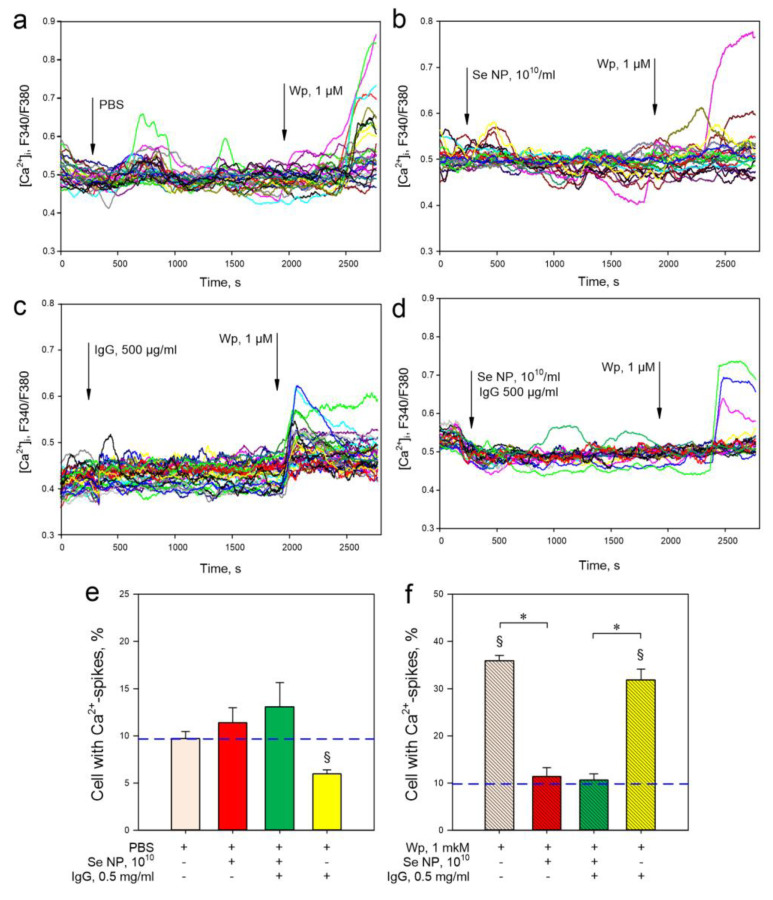
The assessment of the proportion of granulocytes with calcium spikes (responses). Examples of cell responses (~30) in randomly taken areas of the visual field with the background of PBS (control) (**a**), SeNPs (**b**), a combination of SeNPs and IgG (**c**) and IgG (**d**) followed by the addition of 1 μM Wp. The proportion of cells with calcium responses immediately after supplementation with PBS (control), SeNPs, IgG, or a combination of SeNPs and IgG (**e**). The proportion of cells with calcium responses to 1 μM Wp supplementation after 20 min incubation with indicated agents (**f**). The panels (**e**,**f**) show the average data for all cells in the field of view. In each variant, the study was performed on six cell samples; in each, at least 200 cells were analyzed. Data are presented as a mean ± SE. §—*p* < 0.05 vs. PBS (blue dotted line), *—*p* < 0.05 between indicated variants. The significance of differences was assessed by the Mann–Whitney test. The six independent experiments were carried out for each variant (*n* = 6).

Since both “respiratory burst” and degranulation depend on the level of cytoplasmic calcium [58], we evaluated the effect of SeNPs and their combination with IgG on the calcium activity of granulocytes. In the control, about 10% of granulocytes generated calcium spikes (Figure 8a,e). The addition of selenium nanoparticles, both with and without the addition of antibodies, did not change the number of cells with calcium responses (Figure 8e). IgG supplementation reduced the number of cells with calcium spikes compared to controls (Figure 8e). The addition of the “breathing burst” inducer Wp increased the number of cells with calcium responses three times (Figure 8a,f) compared to the control. The addition of SeNPs reduced the number of cells responding to Wp by up to ~10% (Figure 8b,f). IgG did not affect the calcium response of granulocytes to Wp (Figure 8c,f). The SeNPs + IgG combination had the same effect as SeNPs (Figure 8d,f). Thus, SeNPs can significantly modulate the immunogenic response of granulocytes against the background of IgG induction. It is usually assumed that such effects are associated with the influence on the cellular signaling regulatory mechanisms. For this purpose, the cell expression profile was studied; the main results are presented in Table 3.

Twenty-four hours after the addition of SeNPs at a concentration of 10^10^ mL^−1^, the level of expression of most granulocyte genes changes slightly (within 25–50%) (Table 2). At the same time, the expression of the *HSP90* gene increased threefold, the *SOD2* gene increased fourfold, and the expression of the *NRF2* gene decreased sevenfold. The introduction of SeNPs likely leads to a change in intracellular Red/Ox homeostasis, which leads to a decrease in the level of *NRF2*, the main regulator of antioxidant response gene expression. In addition, the level of *NRF2* can be suppressed by the increased level of *NFkB*, which increases significantly 24 h after SeNPs administration. A significant increase in the level of *IL-6* expression also indicates the possible role of NF-κB. It should be noted that when using a nanoparticle concentration of 10^9^ mL^−1^, changes in expression levels were much less pronounced and often did not differ from control values.

Twenty-four hours after the addition of IgG, the level of expression of most granulocyte genes changes slightly (within 25–50%) (Table 2). At the same time, the expression of the *HSP90* gene increased by 2.5 times, while the expression of the IL6 gene decreased sevenfold, the expression of the *HO-1* gene decreased sixfold, and the expression of the Catalase gene decreased by 2.5 times.

With the joint addition of SeNPs and IgG, the expression level of most granulocyte genes changes after 24 h. The expression level of the *SOD2* gene increased sixfold. The level of *HSP90* increased fourfold. The level of expression of *NFkb*, *IL6* and *Xrcc4* increased by more than twofold. A decrease is observed only in the expression of the *HO-1* gene (approximately twofold). Interestingly, the effect of the combined addition of SeNPs and IgG usually has the sign of the SeNPs effect, though it numerically differs from the effect of SeNPs. For example, the effect of the combined addition of SeNPs and IgG is greater than the addition of SeNPs alone for *SOD2* by 50%, for *NFkb* by 45%, for *IL6* by 60%, for *Xrcc4* and *Xrcc5* by more than 50%. At the same time, when only IgG alone was added to these genes, a decrease in the level of expression was observed. Thus, IgG enhances the effect of SeNPs, while IgG itself leads to a decrease in the expression level. This fact can only be explained by the interaction between SeNPs and proteins.

**Figure 9:**
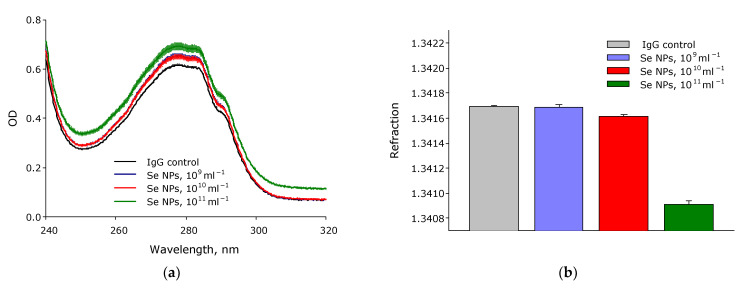

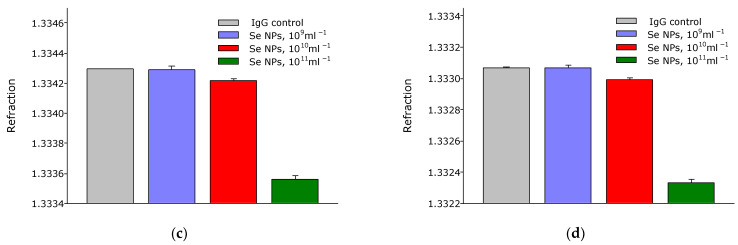
The effect of SeNPs at concentrations 10^9^–10^11^ mL^−1^ on the optical properties of the IgG solution. Optical absorption of protein solutions containing and not containing SeNPs (**a**). Refractometry of protein solutions containing and not containing SeNPs at wavelengths of 435.8 nm (**b**), 589.3 nm (**c**), and 632.9 nm (**d**). The three independent experiments were carried out for each variant (*n* = 3).

The effect of SeNPs at concentrations 10^9^–10^11^ mL–^1^ on the optical absorption of an IgG solution was studied (Figure 9a). It was shown that when SeNPs at a concentration of 10^−9^ mL^−1^ are added to the IgG solution, no change in the absorption of the solution is observed. When SeNPs at a concentration of 10^−10^ mL^−1^ are added to the IgG solution, the absorption in the local maximum at about 280 nm increases by about 5% of the value, although no change in the absorption of the solution is observed at wavelengths of more than 300 nm. When SeNPs at a concentration of 10^−11^ mL^−1^ are added to the IgG solution, the absorption in the local maximum at about 280 nm increases by more than 10% of the value, while at wavelengths of more than 300 nm, an increase in the absorption of the solution by 30–35% is observed.

The effect of SeNPs at concentrations of 10^9^–10^11^ mL^−1^ on the change in the refractive index of the IgG solution was studied (Figure 9b–d). The refractive index was studied under the action of three laser sources with wavelengths of 435.8 nm, 589.3 nm, and 632.9 nm. It is shown that the addition of SeNPs at a concentration of 10^9^ mL^−1^ does not affect the change in the refractive index at all three studied wavelengths. The addition of SeNPs at a concentration of 10^10^ mL^−1^ reduces the refractive index by 6 × 10^−5^–8 × 10^−5^ at all three studied wavelengths. The addition of SeNPs at a concentration of 10^11^ mL^−1^ reduces the refractive index by more than 10^−3^ at all three investigated wavelengths.

**Figure 10:**
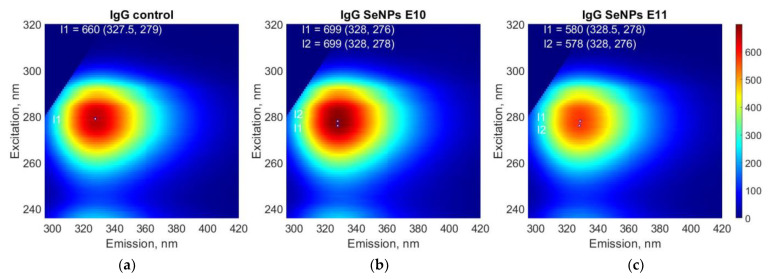
The effect of SeNPs on the fluorescence of IgG molecules. IgG solution (**a**), IgG solution containing SeNPs at a concentration of 10^10^ mL^−1^ (**b**), IgG solution containing SeNPs at a concentration of 10^11^ mL^−1^ (**c**). The three independent experiments were carried out for each variant (*n* = 3).

Since SeNPs at a concentration of 10^−9^ mL^−1^ did not significantly affect the optical absorption and refractive index of protein solutions, then the lowest concentration of nanoparticles used in the study was 10^−10^ mL^−1^. The effect of SeNPs on the fluorescence of IgG molecules was studied (Figure 10). It has been shown that a solution of protein molecules fluoresces most intensely at a wavelength close to 330 nm (327–329 nm) when excited at a wavelength close to 280 nm (278–279 nm). The addition of SeNPs did not significantly affect the shape of the fluorescence regions on the 3D map. The most effective excitation and emission wavelengths did not change. In this case, the addition of SeNPs led to a decrease in the intensity of the fluorescent signal. Thus, when SeNPs (10^10^ mL^−1^) were added, the fluorescence intensity decreased by more than 5%. When the SeNPs were added at a concentration of 10^11^ mL^−1^, the fluorescence intensity decreased by almost 20%.

**Figure 11:**
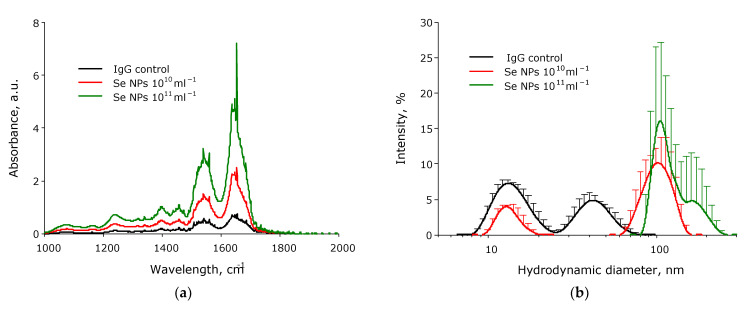
The effect of SeNPs on the change in vibrational–rotational spectra (**a**) and the evolution of nanoparticles in size (**b**) observed in IgG solution. The three independent experiments were carried out for each variant (*n* = 3).

Changes in the vibrational–rotational spectra can be used to assess the interaction of NPs with proteins and even individual cells [59,60,61]. Estimating the hydrodynamic diameter of NPs by the DLS method is one of the standard methods for assessing the interaction of metal NPs with proteins [62,63]. The effect of SeNPs on the change in the vibrational–rotational spectra of aqueous IgG solutions was studied (Figure 11a). It is shown that the addition of SeNPs significantly increases the absorption in the bands of amide I (1650 cm^−1^) and amide II (1550 cm^−1^). When SeNPs (10^10^ mL^−1^) are added, the absorption intensity of the amide I band increased by almost fourfold, and the amide II band by more than threefold. When SeNPs (10^11^ mL^−1^) are added, the absorption intensity of the amide I band increases by almost eightfold and the amide II band by more than sixfold.

The effect of SeNPs on the change in size evolution of particles in aqueous solutions of IgG was studied (Figure 11b). In an aqueous solution of IgG We observed two peaks corresponded to single molecules with an average hydrodynamic diameter of about 15 nm and their aggregates with a size of 40–45 nm. A peak with a size of 100 nm corresponding to SeNPs was also observed. The addition of SeNPs at a concentration of 10^10^ mL^−1^ decreased the amount of individual IgG molecules in the solution by about 40%. The broadening of the peak associated with nanoparticles (the maximum of about 100 nm) was observed. The addition of SeNPs at a concentration of 10^11^ NPs/mL removed a peak of individual IgG molecules. A two-humped peak with maxima at 110–150 nm corresponding to SeNPs was detected in solution at 10^11^ NPs/mL.

## 4. Discussion

According to literature data, the SeNPs of a 50–100 nm size are preferred for use in vivo [16]. Therefore in the present study, we used to control fragmentation to obtain nanoparticles with a rather narrow size evolution (Figure 1 and Figure 2) with a size of 100 nm. SeNPs did not exhibit acute toxicity to primary cultures of fibroblasts and hepatocytes, as well as to the epithelial-like cell line MCF-7 and granulocyte-like culture HL-60 (Figure 3). Weak cytotoxicity at 10^10^ NPs/mL was shown on granulocyte-like culture (Figure 3). It is worth noting that 10^10^ NPs/mL is quite a high concentration. About 100 SeNPs with 100 nm in size will interact with one eukaryotic cell with dimensions of 20 × 20 × 20 μm by calculations. Granulocyte progenitor cells are believed to be much more susceptible to damaging factors [64]. The formation of micronuclei in cells after division is considered a marker of DNA damage [65]. The effect of SeNPs in vivo on the formation of PCE with MN in the murine red bone marrow was studied (Table 2). The genotoxic effect was observed only at a concentration of SeNPs 10^11^ NPs/mL. Thus, SeNPs (exclude concentration 10^11^ NPs/mL) did not show cytotoxicity against that granulocyte-like cell culture, surviving granulocyte culture and myeloid granulocyte progenitor cells.

Granulocytes are a subgroup of white blood cells characterized by a large segmented nucleus and the presence of specific granules in the cytoplasm. The main function of these cells is the generation of ROS and the elimination of pathogens [66]. Overproduction of ROS by granulocytes can lead to pathological damage of host tissues [67]; therefore, ROS production must be precisely regulated. The effects of SeNPs and IgG on the kinetics of ROS generation by murine granulocytes were studied (Figure 4 and Figure 5). We observed an increase in the maximum amplitude of the Wp-induced “respiratory burst” in the presence of antibodies (Figure 4), which can be explained by the IgG-dependent cell priming [68]. The ability of antibodies to enhance ROS generation through FcγR receptors is described in the literature for human neutrophils and granulocytes from murine bone marrow [69,70,71]. On the other hand, SeNPs + IgG reduced the total production of ROS in response to PMA. The decrease of ROS production in response to Wp in the presence of antibodies is also described in the literature on the example of human fibroblasts [72], but the underlying mechanism has not been identified. We proposed several possible scenarios: 1. SeNPs do not interact with IgG and do not change the functional activity of IgG. With such a development of events, the effect of IgG should completely neutralize the effect of SeNPs. 2. IgG molecules completely “cover” SeNPs. As a result, the effect of nanoparticles disappears, but this does not explain why the effect of the combination of SeNPs + IgG is greater than that of IgG alone. 3. IgG molecules interact with the SeNPs, “cover” them and change their properties.

The effect of a combination SeNPs + IgG on the “respiratory burst” depended on the type of activator: in the case of Wp, the maximum amplitude increased; in the case of PMA, both the maximum amplitude and the total production decreased. Wp is a specific ligand for FPR2 (high-affinity receptors of formylated peptides) [73,74]. FPRs are metabotropic receptors associated with G-proteins (G protein-coupled receptor, GPCR). This activation leads to various cell responses, including the assembly of active NADH oxidase on the cell membrane, which is involved in the enhancement of ROS generation [75]. PMA is a direct PKC activator that does not require the participation of membrane GPCR receptors and cytoplasmic calcium [76]. The obtained results suggest that the combination of SeNPs + IgG or IgG alone modulates both the fast receptor-dependent “respiratory burst” phase and the slow “respiratory burst” phase caused by direct PKC activation. This is consistent with literature data on the ability of selenium and IgG to modulate the signal transduction along both the PI3K-dependent pathway (via GPCR) and the PKC-dependent pathway [39,40,41].

In the control, about 10% of granulocytes generated calcium spikes (Figure 6a,e), which is consistent with the data of our previous studies and studies of other authors [47,50,77]. An increase in the concentration of cytoplasmic calcium in response to Wp is well described in the literature and is one of the mechanisms of its activity. Activation of FPRs in response to Wp leads to an increase in the level of cytoplasmic calcium through the following signaling pathway: GPCR activation, PLC-β activation, PIP_2_ hydrolysis to IP_3_, and opening of IP_3_R calcium channels on the endoplasmic reticulum [78,79]. We have found the ability of SeNPs to block Wp-induced calcium responses in mouse granulocytes. Se^+^ cations are capable of inhibiting the IP_3_-dependent release of calcium from the cytoplasmic reticulum [80,81].

In the next phase of the study, we evaluated the effect of SeNPs and the combination of SeNPs on fMLF-induced granulocyte degranulation (Figure 7). We found that SeNPs additionally enhance cell degranulation in response to fMLF. This fact is consistent with the literature data on the ability of selenium to enhance the degranulation of a macrophage cell line [82]. The addition of antibodies did not increase fMLF-induced degranulation; however, this fact is consistent with the literature data on a significant dependence of the effect of IgG on degranulation on the isotype, subtype, and epitope specificity of antibodies [83]. In our case, polyclonal antibodies were used, so we find it difficult to explain more precisely. The combination of SeNPs and IgG had no effect on fMLF-dependent degranulation, as did IgG. Based on this, we suggest that IgGs block the effect of SeNPs on the degranulation of mouse granulocytes.

SeNPs are capable of releasing selenium ions into the surrounding solution, though the concentration of selenium ions should be extremely low. At least, we did not register a decrease in the hydrodynamic radius of nanoparticles or their number with the measurement accuracy available to us during the entire study period. Potentially, selenium ions are able to inhibit the calcium signaling of cells. Thus, selenium ions can inhibit the calcium responses of mouse granulocytes to Wp, while not affecting the Wp-induced ROS production. ROS production is regulated by calcium-dependent and calcium-independent pathways [76,78,79]. SeNPs can likely be considered as a potential inhibitor of calcium-dependent pro-inflammatory pathways, which allow for maintaining the functional activity of immune cells during the treatment of pathological inflammatory processes. Thus, SeNPs are able to significantly modulate the immunogenic response of granulocytes in the presence of IgG. It is usually assumed that such effects are associated with the influence on the cellular signaling regulatory mechanisms. For this, the cell expression profile was studied (Table 3).

The levels of ROS generation by granulocytes affect the expression of genes associated with the development of inflammation. The main pathway is the activation of nuclear transcription factor κB (NF-κB) [84]. This nuclear transcription factor is a pleiotropic regulator of a large number of genes associated with the development of immune and inflammatory responses [85]. This is confirmed by the change in the expression level of genes involved in regulating inflammation: *TNF-α, IL6*, and *NFkB*. The increased level of NF-κB suppresses the expression of *NRF2*, as shown earlier [86]. This explains the decrease in the expression level of genes responsible for the expression of antioxidant enzymes, except for *SOD2*. It is possible that a decrease in *SOD2* expression is associated with the level of *HSP90* expression, which, in turn, may also be associated with a change in NF-κB expression [87]. It is known that an increase in the level of *TNF*-α expression occurs when exposed to SeNPs, and depends on the concentration of nanoparticles and exposure time [88]. Selenium can decrease pro-inflammatory gene expression levels by inhibiting MAP kinase pathways [89]. The addition of the total fraction of antibodies and antibodies with SeNPs to the cells also significantly affected the expression profile of many genes. Often, IgG enhances the effect of SeNPs, while IgG itself leads to a decrease in the level of gene expression (Table 3). The interaction between SeNPs and proteins can only explain this fact. It should be recalled that the data on the respiratory burst could simply explain the interaction of nanoparticles and protein molecules.

The interaction of antibody molecules with SeNPs was analyzed with several optical methods. The addition of SeNPs increased the optical density of IgG solutions (Figure 7a). On the one hand, optical density increases in the absorption region of aromatic amino acid residues, mainly tryptophan. On the other hand, optical density increases in the long-wave region (after 310 nm). The obtained data indicate a change in the protein structure [90] associated with partial denaturation, chemical modification, aggregation, and interaction with the nanoparticle [91]. 

Partial denaturation must lead to a significant increase in the number of water molecules in the hydration shell of the protein. An increase in the number of water molecules in the protein hydration shell leads to a change in the refractive index [92]. The refractive index was measured with high accuracy at three wavelengths (Figure 7b–d), and only at a concentration of 10^11^ SeNPs per ml has recorded a decrease in the refractive index by 0.005. The fluorescence of a solution containing antibodies and SeNPs was studied (Figure 8). The fluorescence excitation maximum of aromatic amino acids falls within the range of 275–283 nm [93], and we recorded a maximum fluorescence intensity of 276–279 nm. Adding SeNPs to IgG decreased the fluorescence intensity and did not change the emission maximum. This indicates that SeNPs can probably induce insignificant degradation of aromatic amino acid residues. Data on the absorption spectra, refractive index and fluorescence spectra of IgG after a significant change in the tertiary and secondary structure during heating at 90 °C 5 min were shown in Appendix A.

The shape of the fluorescence spots on 3D maps also does not significantly change. It may indicate that the chemical modification of the fluorophore does not occur. The FTIR data indicate (Figure 9a) that the addition of SeNPs leads to an increase in absorption in the amide I (1650 cm^−1^) and amide II (1550 cm^–1^) bands. This may indicate partial melting of the protein structure and its aggregation. Using the dynamic light scattering method, the evolution of the sizes of light-scattering particles in an aqueous solution of SeNPs and IgG was studied (Figure 9b). SeNPs at a concentration of 10^10^ mL^−1^ decreased the number of individual IgG molecules. The individual IgG molecules are not observed in the solution at SeNPs, a concentration of 10^11^ mL^−1^. No shift of the peak of individual molecules to the region of smaller sizes is observed after the addition of nanoparticles; therefore, we assume that the polypeptide chain was not damaged. The proportions of light scattering intensity of peaks of individual protein molecules and aggregates changed, but the peaks’ positions did not change. Formation of the IgG aggregates not associated with nanoparticles was not observed., The right shoulder, with a size of about 150 nm, appears in the size distribution of IgG at SeNPs at a concentration of 10^11^ PNs/mL. According to the calculations, it is a projection of a dimer of nanoparticles covered with antibodies ((110 nm + 110 nm + 220 nm)/3 = 146 nm) [94].

Thus, it can be stated that antibodies interact with SeNPs. The antibodies bind to the surface of SeNPs and are present in solutions in such a bound form. At the same time, in a significant part of the antibodies, a partial change in the tertiary and secondary structure is observed. Based on obtained results, we proposed that the binding of antibodies and SeNPs can modulate properties of each other. A combination of SeNPs and IgG can change granulocyte physiology (ROS generation, degranulation and calcium activity) and the level of expression of inflammation and stress-associated genes.

## 5. Conclusions

The SeNPs’ cytotoxicity and ability to modify the functional responses of granulocytes were studied. The SeNPs did not show acute toxicity to fibroblasts, hepatocytes and epithelial-like cell line L-929. Weak cytotoxicity was observed on a granulocyte-like culture of HL-60. The addition SeNPs modified ROS production by murine bone marrow granulocytes and modulated the proportion of granulocytes with calcium spikes. SeNPs increased fMLF-induced degranulation. IgG blocked this effect. Se NPs increase the expression of “stress-associated” genes. Antibodies enhance the effect of SeNPs, while IgG without SeNPs decreases the “stress-associated” gene expression levels. The interaction between SeNPs and antibodies can explain this fact. We established that IgG interacts with SeNPs. Antibodies bind to SeNPs surface in aqueous solutions. A partial change in the tertiary and secondary structure is observed in a significant part of the antibodies. We propose that the interaction of SeNPs with IgG may has potential application in the modulation of immune cell (granulocyte) activity during pathology states.

## Figures and Tables

**Table 1:** Oligonucleotides used for qRT-PCR.

	Genes	GenBank Accession	Oligonucleotide 5′-3′	Amplicon Size, bp
1	*Actb*	NM_007393.4	CCTTCCTTCTTGGGTATGGAATCC	115
			CACCAGACAGCACTGTGTTGGCA	
2	*HSP90*	NM_011631	GTCCGCCGTGTGTTCATCAT	168
			GCACTTCTTGACGATGTTCTTGC	
3	*KEAP-1*	NM_016679	TGCCCCTGTGGTCAAAGT	104
			GGTTCGGTTACCGTCCTGC	
4	*NF-kb*	NM_008689	CCACGCTCAGCTTGTGAGGGAT	106
			GGCCAAGTGCAGAGGTGTCTGAT	
5	*NRF2*	NM_010902	CTCGCTGGAAAAAGAAGTG	240
			CCGTCCAGGAGTTCAGAGG	
6	*Catalase*	NM_009804	AGCGACCAGATGAAGCAGTG	181
			TCCGCTCTCTGTCAAAGTGTG	
7	*SOD2*	NM_013671	GCGGTCGTGTAAACCTCAT	240
			CCAGAGCCTCGTGGTACTTC	
8	*Prx6*	NM_007453	TAAGGACAGGGACATTTCCATCC	145
			CCGTGGAGTTAGGGTAGAGGA	
9	*Xrcc4*	NM_028012	GAGACACCGAATGCAGAAGA	121
			GGTGCTCTCCTCTTTCAAGG	
10	*Xrcc5*	NM_009533	GAAGAACAGCGCTTCAACAG	92
			TCCTGAACAACAATTTCCCA	
11	*LigIV*	NM_176953	ATGGCTTCCTCACAAACTTCAC	103
			TTTCTGCACGGTCTTTACCTTT	
12	*TNFa*	NM_013693	ATGAGAAGTTCCCAAATGGC	125
			CTCCACTTGGTGGTTTGCTA	
13	*AP-1*	NM_010591	CACGGAGAAGAAGCTCACAA	126
			ACTTGTTACCGGTCCTCTGG	
14	*Ki67*	NM_001081117	ATCATTGACCGCTCCTTTAGGT	104
			GCTCGCCTTGATGGTTCCT	
15	*IL6*	NM_031168	TAGTCCTTCCTACCCCAATTTCC	76
			TTGGTCCTTAGCCACTCCTTC	

**Table 2:** The effect of SeNPs administered intravenously on the formation of PCE with MN in the red bone marrow of mice.

SeNPs, NP/mL	Number of Animals	Number of Cells	Number of Cells with MN	Percentage of Cells with MN
0	5	12560	69	0.55 ± 0.06
10^9^	5	12758	73	0.57 ± 0.07
10^10^	5	12931	81	0.62 ± 0.06
10^11^	5	12652	90	0.71 ± 0.07 *

*—a statistically significant difference from the control group (*p* < 0.05). MN—micronucleus.

**Table 3:** The changes in the expression of some “stress” genes in the culture of granulocytes. SeNPs and IgG administration affect the change in the number of mRNA copies of several genes after 24 h. The changes in the amount of mRNA of the most variable genes (*p* < 0.05) in comparison to the level of expression of the same genes in the control are shown. Data represented as a mean ± SE. The five independent experiments were carried out for each variant (*n* = 5).

Genes	Relative Gene Expression			
	Control	Se NPs	IgG	Se NPs + IgG
*HO-1*	1	0.64 ± 0.03	0.15 ± 0.01	0.48 ± 0.02
*HSP90*	1	3.03 ± 0.58 *	2.35 ± 0.51 *	3.83 ± 0.78 *
*NFkb*	1	1.80 ± 0.11 *	0.67 ± 0.01 *	2.60 ± 0.22 *
*NRF2*	1	1.43 ± 0.17 *	0.93 ± 0.04 *	0.71 ± 0.06 *
*Catalase*	1	1.19 ± 0.12 *	0.42 ± 0.00 *	1.61 ± 0.12 *
*SOD2*	1	4.06 ± 0.34 *	0.94 ± 0.01 *	6.06 ± 0.17 *
*Prx6*	1	0.09 ± 0.01 *	0.14 ± 0.02 *	0.14 ± 0.01 *
*Xrcc4*	1	1.56 ± 0.12 *	0.87 ± 0.03 *	2.30 ± 0.06 *
*Xrcc5*	1	1.08 ± 0.13	0.92 ± 0.02	1.65 ± 0.15
*TNF-α*	1	1.72 ± 0.12 *	0.94 ± 0.01	1.06 ± 0.11
*AP-1*	1	1.12 ± 0.16	0.65 ± 0.06	1.41 ± 0.10
*Ki67*	1	0.67 ± 0.02 *	0.76 ± 0.02 *	1.02 ± 0.13
*IL6*	1	1.27 ± 0.12 *	0.14±0.01 *	2.00 ± 0.24 *

*—*p* < 0.05 vs. control. The significance of differences was assessed by the Mann–Whitney test. The six independent experiments were carried out for each variant (*n* = 5).

**Figure A1:**
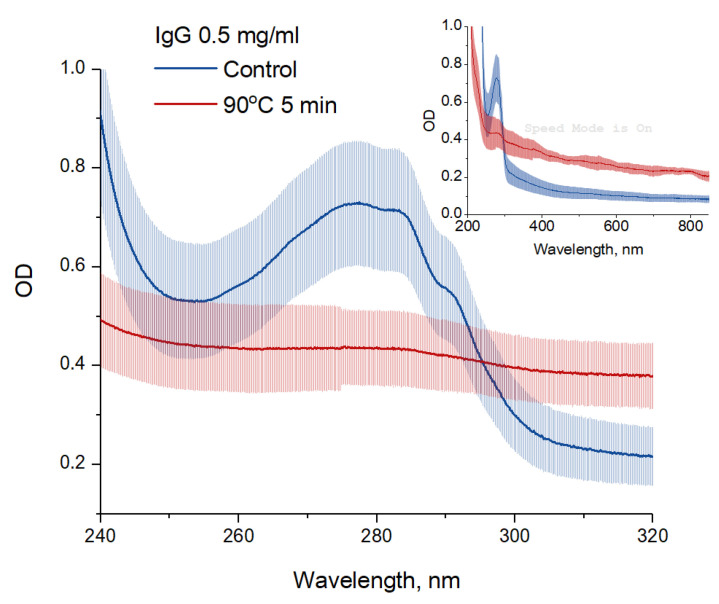
Optical absorption spectra of IgG 0.5 mg/mL solution before (blue) and after 5 min incubation at 90 °C (red). Measurements were carried out at 22 °C. Data are presented as a mean ± SE. The three independent experiments were carried out for each variant (*n* = 3).

**Figure A2:**
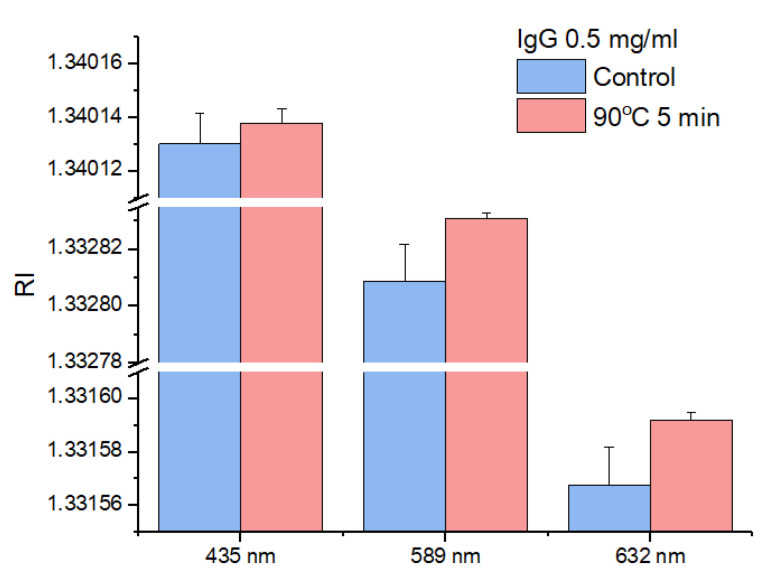
Refractometry of IgG 0.5 mg/mL solution before (blue) and after 5 min incubation at 90 °C (red). Measurements were carried out at 22 °C. Data are presented as a mean ± SE. The three independent experiments were carried out for each variant (*n* = 3).

**Figure A3:**
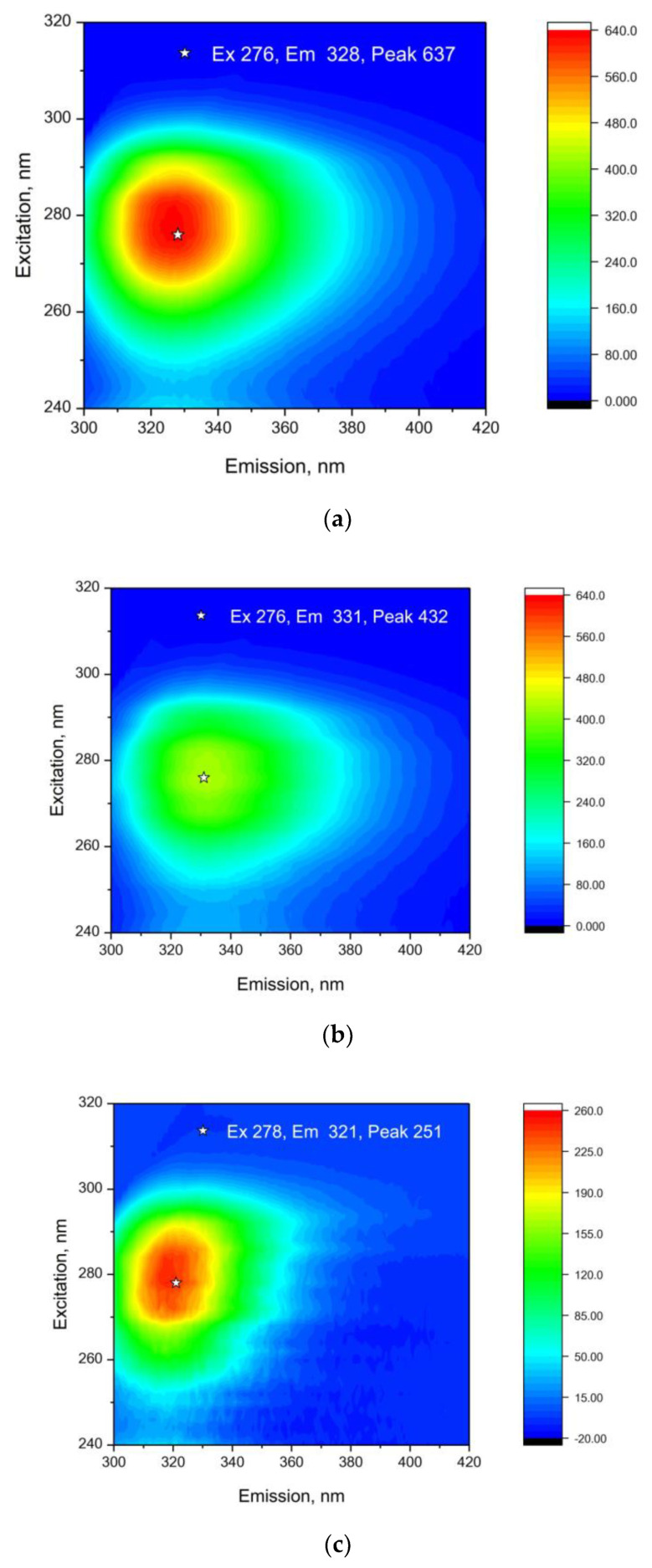
3D fluorescence spectra of IgG 0.5 mg/mL solution before (**a**) and after 5 min incubation at 90 °C (**b**). (**c**) Differential spectrum between subfigure (**a**,**b**). Measurements were carried out at 22 °C.

## Data Availability

The raw data supporting the conclusions of this article will be made available by the authors without undue reservation.

## Appendix A

Data on the absorption spectra, refractive index and fluorescence spectra of IgG before and after a significant change in the tertiary and secondary structure were also received. We used denaturation by heating at 90 °C 5 min as a positive control. Denaturation dramatically decreased the absorption of IgG solution in the region of 280 nm and increased absorption in the region after 300 nm (Figure A1). A decrease in the refractive index of about 0.01 was observed after heating (Figure A2). This is three orders of magnitude higher than when IgG binding to NPs. Denaturation also changed the fluorescence spectrum of the IgG solution and reduced the fluorescence intensity at the 276/330 nm peak by ~30% (Figure A3).
